# How Do People with Multiple Sclerosis Experience Prognostic Uncertainty and Prognosis Communication? A Qualitative Study

**DOI:** 10.1371/journal.pone.0158982

**Published:** 2016-07-19

**Authors:** Laura Dennison, Ellen McCloy Smith, Katherine Bradbury, Ian Galea

**Affiliations:** 1 Academic Unit of Psychology, University of Southampton, Southampton, United Kingdom; 2 Faculty of Medicine, University of Southampton, Southampton, United Kingdom; 3 Clinical Neurosciences, Clinical and Experimental Sciences, Faculty of Medicine, University of Southampton, Southampton, United Kingdom; University of Stirling, UNITED KINGDOM

## Abstract

**Background:**

Disease progression in multiple sclerosis (MS) is highly variable and predicting prognosis is notoriously challenging. Patients’ prognosis beliefs, responses to prognostic uncertainty and experiences of prognosis-related communication with healthcare professionals (HCPs) have received little study. These issues have implications for patients’ psychological adjustment and are important in the context of the recent development of personalised prognosis forecasting tools. This study explored patient perspectives on the experience of prognostic uncertainty, the formation of expectations about personal prognosis and the nature of received and desired prognosis communication.

**Methods:**

15 MS patients participated in in-depth semi-structured interviews which were analysed using inductive thematic analysis.

**Results:**

Six themes captured key aspects of the data: *Experiencing unsatisfactory communication with HCPs*, *Appreciating and accepting prognostic uncertainty*, *Trying to stay present-focused*, *Forming and editing personal prognosis beliefs*, *Ambivalence towards forecasting the future*, and *Prognosis information delivery*. MS patients report having minimal communication with HCPs about prognosis. Over time MS patients appear to develop expectations about their disease trajectories, but do so with minimal HCP input. Provision of prognosis information by HCPs seems to run counter to patients’ attempts to remain present-focused. Patients are often ambivalent about prognosis forecasting and consider it emotionally dangerous and of circumscribed usefulness.

**Conclusions:**

HCPs must carefully consider whether, when and how to share prognosis information with patients; specific training may be beneficial. Future research should confirm findings about limited HCP-patient communication, distinguish predictors of patients’ attitudes towards prognostication and identify circumstances under which prognostic forecasting benefits patients.

## Introduction

Multiple sclerosis (MS) is a chronic, degenerative disease of the central nervous system. It is the leading cause of non-traumatic neurological disability in young adults [1], and can produce an assortment of symptoms including visual disturbance, spasticity, speech distortions, bladder and bowel dysfunction, fatigue, pain and cognitive impairment. MS is also renowned for having a variable and uncertain trajectory. The vast majority of people with MS (pwMS) initially experience a relapsing-remitting phase; with around 80% of these converting, eventually, to a progressive phase where disability accumulates [2]. Despite the identification of prognostic principles [3] accurate prediction of an individual patient’s short and long-term disease activity, progression and ultimate disability level remains challenging.

Clinical guidelines (e.g. from the National Institute of Clinical Excellence [4]) emphasise the importance of open and honest communication and information provision for pwMS but make no specific recommendations about how an individual’s likely prognosis and the inherent uncertainty surrounding disease outcomes is communicated. There is also little research regarding this communication. Clinical experience suggests that communication practices are probably highly variable, with each treating physician making an experience-based clinical judgement about their individual patient’s disease trajectory and then determining whether, when and how to communicate expectations and predictions to the patient. Heesen et al.[5] found that amongst mild-moderately affected pwMS from a German neurology clinic, very few (6%) had ever received prognostic counselling, although interestingly only a minority (11%) wanted more prognosis information than they had received. Other German research with severely affected patients uncovered dissatisfaction; most respondents considered communication about disease progression to be important but felt that doctors inadequately addressed it [6]. More generally, the unmet information needs of pwMS, especially at the time of diagnosis, are well-documented [7,8].

It is not surprising that uncertainty, or a lack of clarity or predictability, is noted to be a common experience for pwMS and their families; the uncertain and ambiguous nature of the disease and the implications of this for patients and their families has received substantial research attention [9]. Although the meaning of the term ‘uncertainty’ is not always clearly operationalised or explicitly defined in the MS literature, influential theoretical work by Mishel [10] defined illness uncertainty as multifactorial and encompassing complexities, doubts, and unpredictability relating to symptoms, diagnosis, treatment, relationships, disease progression and future planning. Overall, research in MS suggests that experiencing illness uncertainty contributes to emotional distress and the practical challenges associated with living with the disease. A review established that patients who feel uncertain or lack a coherent understanding of their MS tend to score higher on psychological distress measures than those who consider themselves to have a clear understanding of their disease[11]. Indeed patients’ understanding and perceptions of their condition are prominent factors within several key psychological models of adjustment to chronic illnesses (e.g. [11,12]) with highly negative, fearful or incoherent illness perceptions viewed as contributing to distress and maladjustment. Furthermore, qualitative research highlights day-to-day and longer-term uncertainty as being central to the experience of living with MS and problematic to come to terms with [13,14]. Importantly, however, existing work on uncertainty in MS has not focused in on any specific source of uncertainty and has typically employed questionnaire measures of uncertainty that have been developed in line with Mischel’s [10] multifactorial conceptualisation. Research specifically addressing the experience and impact of *prognostic* uncertainty in pwMS is lacking.

### Aims and research questions

The current study explores how pwMS experience prognostic uncertainty and communication with HCPs about prognosis. It aims to understand how prognostic uncertainty affects pwMS, if and how they form expectations about their disease trajectory and what communication they receive and want. These topics are both interesting in their own right and timely to investigate given recent developments of an online analytical processing tool (OLAP) which supports individualised prognostic risk forecasting in MS [5,15,16]. The latest version of the tool supports 30 year forecasting by predicting time to milestones including conversion to secondary progressive disease, EDSS 6 (i.e. walking using a stick), EDSS 8 (i.e. perambulated in wheelchair) and EDSS 10 (i.e. death from MS)[16]. Current work with this tool focusses on its use to support shared treatment decision-making. However, the availability of personalised prognostic information has repercussions beyond the realm of treatment decision-making and could influence the psychosocial adjustment of pwMS and their families. Initial research using an earlier (3 year forecast) version of the OLAP tool suggested that prognosis forecasting was understandable and acceptable to patients[5]. However, it remains unclear what psychological benefits (e.g. reduced emotional distress following a reassuring prognosis estimate) or harms (increased distress and hopelessness following a more pessimistic prognosis estimate) the tool could elicit, whether pwMS would welcome its use, and under what circumstances it would prove relevant and useful. For these reasons we embarked on an investigation of pwMS’s views and experiences of prognostic uncertainty and prognosis communication. The aim was to provide insight into the context in which prognosis prediction and communication aids such as the OLAP tool would operating in, and the range of possible consequences of encouraging HCPs to consider and discuss prognosis more frequently, extensively and openly.

## Method

### Data collection

This study was approved by the University of Southampton ethics committee. Data was collected between Spring 2014 and Summer 2015 via semi-structured telephone interviews with pwMS. Telephone interviews were chosen to allow participants from a wide geographical area (across the UK) to participate, to limit burden on pwMS and to ensure that mobility limitations, MS symptoms, work or family commitments did not preclude participation. Although some researchers consider face-to-face interviews superior for establishing a rapport with participants when researching emotive topics, we have previously successfully used telephone interviews to elicit deeply personal, rich and detailed accounts of illness experiences in pwMS [17,18].Choosing telephone interviews over face-to-face interviews allowed us to connect with pwMS who would have found a face-to-face meeting either impracticable or intimidating.

Five of the interviews were conducted by LD (an academic health psychologist with substantial experience in qualitative research) and 10 were conducted by EM (a medical student). EM had practical training in qualitative interviewing and ongoing supervision from LD throughout data collection. A schedule of open-ended questions (S1 Fig) encouraged participants to discuss their understanding of how MS might affect them over the longer-term, how they had reached their current understanding, how any uncertainty about prognosis affected their life, whether and under what circumstances they would want more information about their likely prognosis, and anticipated consequences of receiving this information. Participants were encouraged to discuss issues they considered to be personally important and prompts were used to further probe topics they spontaneously raised. The interview schedule was developed by LD and EM with input from IG. Following its first use minor edits to wording and question order were made to improve its comprehensibility and relevance. A series of brief questions were used to ascertain demographic and clinical characteristics. Interviews lasted between 20 and 78 minutes (mean 46.5) and were audio-recorded and transcribed verbatim.

### Participants

Inclusion criteria were a diagnosis of MS and age ≥18 years. An overview of the study was disseminated via the UK MS Society website and social media and through emails to members of the UK MS Register. PwMS who were interested in participating responded to the researchers directly. Participants received information sheets and signed consent forms prior to enrolment. 23 people expressed interest in the study. We begun by interviewing all participants that consented but as data collection (and concurrent data analysis) progressed we deliberately sampled from the pool of available participants to ensure the final sample varied in terms of MS course (relapsing remitting, primary and secondary progressive), time since diagnosis, age and gender. Purposive sampling was possible because participants typically disclosed this information to the researcher at the point of expressing interest or of returning the consent form. At later stages of the data collection we also made deliberate efforts to attract interest from younger and more newly diagnosed pwMS by editing the recruitment adverts/information to make it clear that we were especially interested in hearing from people with these characteristics. Once we began purposive sampling we thanked those that consented for their interest and willingness to be involved and advised them that we were nearing the end of the project and narrowing our focus in order to explore the experiences of participants with particular profiles. Where relevant we advised volunteers that their participation would not required and offered them the opportunity to receive a summary of the research results. Recruitment ceased after 15 interviews as by this point we had succeeded in collecting data from a diverse sample and analysis had reached the point where no new themes were occurring (i.e. saturation). Participants received a £15 retail voucher to thank them for their time.

Table 1 shows participant characteristics. Fifteen participants were interviewed (twelve (80%) female). Age ranged from 31 to 68 (median 48 years). Eight participants identified themselves as having relapsing-remitting MS, four secondary progressive and three primary progressive. Time since diagnosis ranged from 2 months to 22 years (median 9 years). The clinical and sociodemographic characteristics of the 15 participants broadly map onto the characteristics of UK MS patients including the high (2.4 to 1[19]) female to male gender ratio.

**Table 1:** Participant Characteristics.

	Median (range) or N (%)
**Gender** Female Male	12 (80%) 3 (20%)
**Age**	48.0 (31–68 years)
**Marital status** Single Co-habiting Married Divorced	6 (40%) 2 (13.3%) 6 (40%) 1 (6.7%)
**Type of MS^1^** RR SP PP	8 (53.3%) 4 (26.7%) 3 (20%)
**Time since Diagnosis (years) ^1^**	9.0 (2 months- 22 years)

^1^Self-reported by participants

### Analysis

The data was analysed using thematic analysis [20], a systematic, robust method for developing rich, detailed accounts of qualitative data. LD undertook the analysis; EM and KB gave input into emerging themes and coding manuals. The analysis was inductive, meaning that it was built upwards from the data, rather than originating from a pre-existing theoretical framework or researcher expectations. Recordings and transcripts were listened to and read repeatedly in order to immerse the researcher in the data. NVivo software (v10) was used to support coding of the data. Initial coding was then undertaken whereby descriptive labels were attached to text segments which contained salient material relating to the research questions. Analysis proceeded to develop themes from these ‘codes’ by clustering together similar codes. Analysis commenced after ten interviews had been completed. Further data collection then took place, purposively sampling younger and more recently diagnosed participants. This concurrent data collection/analysis strategy allowed us to elaborate on emerging themes and created a more rigorous analysis since we deliberately sought data that might not fit with our identified patterns and accounts. Themes were progressively reviewed, refined, organised and relabelled until a set of rich, coherent themes and subthemes describing key aspects of the data was produced.

## Results

The analysis identified 6 key themes which incorporated 21 subthemes (see Table 2). In the following sections, the themes are described and illustrated using quotations from participants (anonymised, and labelled according to age, gender, MS type and years since diagnosis). Except where specifically stated, the analysis did not reveal different patterns of findings that could be clearly related to participants clinical or sociodemographic characteristics.

**Table 2:** Themes and Subthemes.

Theme	Subtheme
Experiencing unsatisfactory HCP communication	• The legacy of difficult diagnosis experiences • Limited and/or unexploited opportunities for discussing prognosis/progression • Perceived differences between neurologists and nurses
Appreciating and accepting prognostic uncertainty	• Understanding the variability of progression and prognosis in MS • Philosophical/spiritual acceptance of uncertainty
Trying to stay present-focussed	• Not looking ahead as a coping strategy • Mind wandering into dangerous territory
Forming and editing personal prognosis beliefs	• Coming to one’s own conclusion about likely prognosis • Exposure to other pwMS • Minimal & vague HCP input • Finding things out for oneself • Growing experience with own disease
Ambivalence towards forecasting the future	• Indecision and contradiction • Assumption that forecast would be bad news • Danger of demoralisation and despair • Usefulness of information
Prognosis information delivery	• Timing and receptiveness • Personality differences • Authority and expertise • Setting • Ongoing support vs one-off discussion

### Experiencing unsatisfactory HCP communication

Participants’ accounts of interactions with HCPs about their prognosis frequently digressed to detailed emotional accounts of negative communication and support experiences around the time of diagnosis.

I was left over the weekend with no knowledge, no ability to contact anyone and spent most of the weekend on the phone to the Samaritans. (P12: Female, 44, RRMS, 1yr)

His lack of communication was just really upsetting. (P13: Female, 42, RRMS, 2mths)

Distress relating to diagnosis experiences clearly persisted over time and appeared to create negative expectations and perceptions of future interactions with doctors (particularly neurologists). Neurologists tended to be viewed as ‘diagnosers’ with little time and interest in patients’ ongoing struggles. In contrast, MS nurses tended to be perceived as ideally situated for discussing emotional and practical issues relating to MS. Participants tended to be positive about the communication and support they received from nurses although they did not all have access to a nurse and were conscious that the valued supportive role nurses could provide was limited by their workload.

MS nurses are the ones who generally are the best ones to talk to you face-to-face. (P10: Female, 51, RRMS, 10yrs)

All participants perceived that opportunities to discuss their prognosis had been limited. In some cases this appeared to reflect infrequency of contact with HCPs, the brief nature of consultations and abrupt clinician (especially Neurologist) communication style. In other cases, the participant deliberately held back questions because they did not feel ready to hear the answers. This sense of not being ready and wanting to ‘hold back’ appears to relate to the ‘Trying to stay present-focused ‘ theme described in more detail below.

I didn’t want to know about it that much anyway, because I was just trying to absorb it all. (P12: Female, 44, PPMS, 2yrs)

### Appreciating and accepting prognostic uncertainty

Participants clearly comprehended MS to be a variable disease with inherent uncertainty. Participants were tolerant of the fact that HCPs did not have all the answers and could not confidently predict how MS would affect them over time

I know due to the nature of MS … that they probably couldn’t answer all of the questions…a lot of the questions that I have had. (P13: Female, 42, RRMS, 2mths)

Along with theoretical ‘knowing’ that MS prognosis is uncertain, several participants described philosophical stances towards accepting an inability to know or influence the future. This perspective was described as originating from religious belief or as an outcome of undertaking psychological therapy

I recognise open answers are a reality in the whole of life. (P11: Female,48, RRMS, 1yr)

### Trying to stay present-focussed

A ubiquitous strategy for living with an uncertain but threatening future was to stay in the present as much as possible. “*l I just try to live one day at a time”(P3*: *Female*, *65*,*SPMS*, *3yrs)”*, *“take it as it comes” (P12*: *Female*, *44*, *PP*, *2yrs) and “get on with it [life]” (P2*, *Male*, *48*, *PPMS*, *20yrs & P11*, *Female*, *48*, *RRMS*, *1yr)*, *“try not to think about it too much” (P10*, *Female*, *51*, *RRMS*, *14yrs)*. This approach appeared to be based on the wisdom that *“whatever will be will be” (P1*, *Female*, *45*, *RRMS*, *16yrs &P13*, *Female*, *42*, *RRMS*, *2mths)* and that “*if the worst does happen*, *I’ll deal with that as and when” (P13*, *Female*, *42*, *RRMS*, *2mths)*. This present-oriented approach appeared to inhabit a spectrum ranging from those who had extensively considered future scenarios then concluded that their time/energy is best spent dealing with day-to-day problems and pleasures, to those who (aware or not) avoided thinking about the future because they feel unable to face it.

It’s not wanting to realise what the, it’s realising that things could get worse or that things, that things will get worse but it’s hiding away, it’s more comforting. (P2: Male, 48, PPMS, 20yrs)

I refuse to think about what is going to happen, I don’t like that I’m afraid. (P8: Female, 53, SP, 17yrs)

Despite attempting to be present-focussed, participants inevitably experienced thoughts and worries about future disability and dependence. For instance, one participant (P3: Female, 65, SP, 9yrs) described a deliberate present-oriented approach to coping with MS but also revealed intrusive, distressing future-related thoughts about becoming dependent on others and spoke of how she had contemplated assisted suicide if such circumstances arose.

### Forming and editing personal beliefs about prognosis

Despite discussing the inherent uncertainty surrounding MS progression and declaring that they did not know their likely prognosis, most participants nevertheless described well-formed personal beliefs about their prognosis. Early beliefs seemed to be strongly influenced by salient, emotional images or memories of other pwMS they knew personally, or had seen in the media. Often these individuals had severe, debilitating disability due to MS and accordingly early personal prognosis beliefs tended to be fearful and extreme.

And other people I know have got it are, absolutely, are stricken with it, I’ve got one on the verge of death. (P11: Female, 48, RRMS, 1yr)

[I have] little experience of good stories, mainly bad, and all with the outcome of eventually death, which doesn’t really, reinforce your own situation very well. (P6: Male, 62, PPMS, 21 years)

Early beliefs appeared to evolve over time and be subject to ongoing editing and revision as other sources of information about MS prognosis were encountered. Participants typically describe HCPs as only a minor influence in how their prognosis understanding came about. Importantly though, HCPs communication appeared to attenuate or moderate early fearful beliefs about prognosis by providing verbal explanations about the variability of outcomes and the non-necessity of progression and/or severe decline.

I immediately thought it was a degenerative disease because I didn’t know anything about it. Umm… and he [the neurologist] said ‘not so, necessarily’. (P12: Female, 44, PPMS, 2yrs)

Some participants described how their neurologists had given indications that their MS was either progressing slowly or not highly active. This communication–tentatively positive yet vague- was frequently the only conversations about prognosis participants remembered having with HCPs. Others claimed to have never received information about their likely prognosis from HCPs.

But the prognosis I think was quite vague. (P13: Female, 42, RRMS, 2mths)

I don’t think I was told anything about how it would progress. (P2: Male, 48, PP, 20yrs]

In the absence of clear, timely and detailed information about likely prognosis from HCPs most participants described that in the months or years after diagnosis they undertook extensive personal research, using various sources including the internet, journals and MS organisations. They recognised the variable quality of information and were sometimes frustrated by the lack of personally tailored information. They also often described consciously avoiding very negative information and seeking out positivity.

Another influence on the development of prognosis beliefs was the individual’s growing experience with their own disease.

I know it’s very slow for me. (P11: Female, 48, RRMS, 1yr)

Participants who had several years or decades of MS experience appeared to base their understandings of future likelihoods on their experiences to date (including looking back with hindsight at pre-diagnosis disease activity). This meant that pwMS who were many years post-diagnosis or post symptom onset expressed more confidence about how they expected their disease to affect them than those more recently diagnosed. People tended to believe that future experiences would mirror past experiences.

Going on past time, past diagnosis, the amount of time that I’ve had the MS then it’s more likely now to stay on the same level now for an indefinite period, there shouldn’t be any rapid decline or rapid improvement. (P2: Male, 48, PPMS, 20yrs)

I’ve got experience of it in the last 5 years. It’s accelerating. (P6: Male, 62, PPMS, 21 yrs)

I can clearly see there is a long slow deterioration. But it’s twenty years I am looking at. (P11:, Female,48, RRMS, 1yr)

### Ambivalence towards forecasting the future

Participants were often unable to settle on a position about whether they wanted more insight into their likely long-term prognosis. Participants changed their minds backwards and forwards during the interview and demonstrated internal conflict.

I kind of presumed that it wasn’t actually possible to … to tell. But God if it was possible I would really like to know. I think… Oh God, or do I? Actually yes I would, of course I would. (P13: Female, 42, RRMS, 2mths)

Oh I don’t know. Maybe, I don’t know, Yeah I think so, I’d like to know. (P2: Male, 48, PPMS, 20 yrs)

Would that be useful? You know this? That’s a real toughy, I’ve never even thought of that before. Would I want to know? Um, yeah I think I would, I think I would but I’m not 100%. It’s a toughy though isn’t it? (P1: Female, 45, RRMS, 16yrs)

Because participants were not pressed to arrive at a final, unambiguous answer during the interviews, reporting frequencies “for” and “against” more prognosis information is not straightforward. However, considering overall interview content, five participants were broadly classifiable as wanting more information, six as not wanting more information, and four as very unsure. No relationships between information preferences and disease type (progressive vs. relapsing-remitting), disease duration, gender or age were apparent.

The hypothetical nature of accurately predicting MS progression appeared to compound the participants’ difficulties in articulating their views, as did the challenge that they would only be able to judge the likely impact once they had heard whether their prognosis was positive or negative.

Often, participants appeared to assume that information about the future would be bad news. A dominant concern was that negative information would elicit hopelessness and demoralisation.

If the future is really bad and really bleak then if I knew about that now then I suppose I could be tempted to, to just, give up now or something. (P2: Male, 48, PPMS, 20yrs)

If I had to think about what could happen, well then, you know, you might as well shoot me. (P10: Female, 51, RRMS, 14yrs)

Would I want to know? No I wouldn’t. Because I think it can make you cuckcoo, (P9: Female, 38, RRMS, 8yrs)

I think ignorance is bliss in some ways. (P13: Female, 42, RRMS, 2mths)

Those that had already experienced moderate to severe disability believed that mental adaptation to a slow, creeping progression seemed easier to handle than knowing what one must face in advance.

I find that the changes are so gradual and over time that you kind of, each little thing that happens you get used to it, before something else will happen. It’s not happening all at once, each change is gradual and easier to deal with that way. (P8: Female, 53, SPMS, 17yrs)

Perceived usefulness of prognosis information appeared pivotal in whether participants would want to receive it. Often the information was not considered useful as participants did not believed it would change anything for them.

If somebody knew something was going to happen but they knew how to treat it, then I would want to know, then you’d want the treatment. But if there is no treatment and there is nothing you can do about it then you probably wouldn’t want to know. (P10: Female, 51, RRMS, 14yrs)

No, it’s almost better to just avoid it. I don’t think it makes any difference about knowing. (P11: Female, 48, RRMS, 1yr)

What would you do with that information then? And you can’t change it. Why bother knowing it anyway? (P2: Male, 48, PPMS, 20yrs)

If they could tell me then it wouldn’t make any difference. (P5: Female, 68, SPMS, 13yrs)

Several participants did believe that receiving prognosis information would be useful. Anticipated impacts included prompting them to undertake personally valued activities whilst physically able, taking life easier to try to influence disease outcomes, financial and practical planning and preparing family members.

I would be looking to see if there were any answers, solutions to the problem and if you don’t know then you can’t look for solutions can you. (P10: Female, 51, RRMS, 14yrs)

I might think of moving to a bungalow or something like that. (P10: Female, 51, RRMS, 14yrs)

I would like to know what I am dealing with…. But I would just want to prepare, I suppose the best I can, prepare my children. (P13: Female, 42, RRMS, 2mths)

If I was told I would be in a wheelchair in ten years’ time, there is loads of things I would want to do, before that. (P13: Female, 42, RRMS, 2mths)

### Views about delivering prognosis information

Participants’ believed that HCPs must make challenging judgements about what prognosis information to share and when. Participants suggested that individual and personality differences influenced information preferences.

Some people can understand and take in information a bit more than other people can so they need to be careful about how they tell people. (P2: Male, 48, PPMS, 20yrs)

I think it is just judging the patient. (P12: Female, 44, PPMS, 2yrs)

I think, it’s very hard to know whether someone wants to know, or not,… unless you actually ask the question but then if you say ‘would you like to know?’, I don’t know, it depends on the individual, on the person. (P9: Female, 38, RRMS, 8yrs)

Some participants also described how timing affects receptiveness to prognosis information. Of those who mentioned timing, all felt the point of diagnosis was unsuitable (“*If I’d have been given that info when I was diagnosed I would probably have jumped off a cliff*.*” P12*: *Female*, *44*, *PP*, *2yrs*)). Beyond that, there was no consensus between participants (or indeed certainty within individuals) about what constituted appropriate timing.

There are times when you are ready to hear it and there are times when you are not […] you should be able to read people to know whether they want to know or whether they don’t because sometimes you can say ‘yes ok I’ll listen to it’ and then other times you think ‘oh I don’t want to hear this. (P4: Female, 56, SPMS, 22yrs)

Those that expressed an opinion about the mode of personalised prognosis information delivery favoured face-to-face communication from a HCP and were concerned with privacy and comfort of the setting.

From a professional. A consultant. (P5: Female, 68, SPMS, 13yrs)

Not a letter, not an email. (P1: Female, 45, RRMS, 16yrs)

Relevant expertise was considered vital for prognosis information deliverers but support-giving skills were also considered pertinent and MS nurses were noted for having these.

If I was going to be told about how in the future it will be really bad, I’d want someone who’s an expert who could answer any questions I had. Well I mean, I think that’s all. I wouldn’t mind what status they had but I’d just want them to be knowledgeable. (P3: Female, 65, SPMS, 9yrs)

An MS nurse, because they are very well trained and very empathetic with the situation, to have an in-depth consultation with the person, in a neutral environment, to try and sort of lighten the picture rather than darken it. (P6: Male, 62, PPMS, 21yrs)

Participants emphasised how they felt prognosis information should be communicated not as a one-off conversation but as part of joined-up care that prioritised their emotional wellbeing together with appropriate disease-modifying treatments and symptom management.

They should have people that, other people around and place to support you after you have been told these things. (P2: Male, 48, PPMS, 20yrs)

## Discussion

### Key findings

This study represents the first investigation into the experiences of pwMS regarding prognostic uncertainty and prognosis communication. The analysis suggested that limited communication about prognosis takes place between pwMS and HCPs, a finding which, together with the global dissatisfaction expressed about interactions with HCPs, was not unexpected and concurs with previous research on communication in MS [7,8]. Interestingly though, participants apparently accepted prognostic uncertainty, and were highly ambivalent about the prospect of finding out more. The hesitancy about prognosis information contrasts with previous research characterising MS knowledge as empowering [21,22] but concurs with research suggesting that pwMS work hard to evade the emotional threat of disease progression [18].

In spite of minimal HCP communication and a realistic appreciation of the difficulties in accurate prognostication, we found that pwMS developed beliefs and expectations about their prognosis; particularly about the pace of worsening. The idea of personal beliefs about MS prognosis emerging over time is consistent with the Common-Sense Model of illness perception [23]. This theory’s central tenet is that patients develop ‘lay’ or ‘common-sense’ understandings of their health conditions based on abstract cultural knowledge about the illness (e.g. images of pwMS in wheelchairs), communication from authoritative sources such as HCPs (e.g. about variability) and personal symptom experiences. Common-sense understandings can be misaligned with medical opinion and are not necessarily declared by patients to HCPs. Importantly, research in MS and other diseases shows that regardless of their correspondence to reality, common-sense illness understandings influence coping, psychological and physical health outcomes [24–26].

### Implications for clinical practice

A number of novel findings emerged which should inform improvement of HCP behaviour around discussing and/or delivering prognostic estimates. Firstly, this study highlights the emotive nature of MS prognosis as a topic and indicates that pwMS may require careful preparation and emotional support when this is discussed; referral to psychology services or to patient support groups may be helpful for some patients, particularly given the fact that several participants referred to self-harm, suicide or having mental health difficulties as consequences of getting negative prognosis predictions It also appears that clinicians would benefit from training in skills for discussing prognosis and that the role of MS nurses, as well as neurologists, should not be overlooked. Secondly, current findings demonstrated that although patients are unlikely to welcome detailed prognosis forecasts at diagnosis, early communication from neurologists, (even if brief and vague), about the variability of outcomes and the non-inevitability of wheelchair use is incredibly significant for pwMS, functioning to counter initial extreme and distressing illness perceptions. In early consultations clinicians could also acknowledge that prognosis-related discussions can be overwhelming whilst simultaneously conveying openness to revisiting dialogues when the pwMS feels ready.

Finally, the ambivalence towards receiving forecasts of prognosis demonstrated in this study suggests that HCPs should not assume that all PwMS want to know their prognosis. It was clear that caution and sensitivity need to be exercised if prognosis forecasting tools are introduced into clinical practice and their predictions shared with pwMS. Prognostic calculators appear to be best suited to presentation as part of a clinical consultation or support programme, rather than stand-alone tools.

### Limitations and future directions

A number of limitations to the current study should be noted. The first concerns sampling. We purposively sampled participants with varied clinical and sociodemographic characteristics in order to build up a picture of issues that are relevant for a range of pwMS. However, in accordance with the qualitative methodology, we did not seek to sample representatively. The recruitment approach may have attracted those with strong views about prognosis communication and may have failed to recruit those less engaged with actively managing their MS, and those who use more denial and avoidance based coping strategies. If this was the case we might expect a representative sample to have less appetite for information about their prognosis. This study may also have attracted patients who have had negative experiences and/or limited communication with HCPs and who wanted to share their frustrations about this. If this was the case, our findings might indicate a more negative picture of patient-doctor communication than is warranted.

The suggestion that limited patient-HCP communication about personal prognosis takes place requires replication in a larger sample. It would also be valuable to establish whether the apparent paucity of prognosis communication is a UK phenomenon or more widespread; the limited existing research suggests scarcity might indeed be common [5,6]. Research into HCPs’ views and experiences of prognostication in MS would also help to understand factors influencing prognosis communication.

This study revealed substantial variability in responses to prognostic uncertainty and appetite for information. However, the study design was not suitable for detecting or quantifying associations between particular viewpoints or experiences and patient characteristics (e.g. progressive and relapsing remitting disease subtypes). More research is needed to delineate which pwMS want more prognosis information and to establish the best timing and clinical setting to discuss prognosis. Large questionnaire studies would permit investigation of associations between prognosis information preferences and clinical, sociodemographic and psychological variables.

This study collected only very limited clinical data (MS type and time since diagnosis) and this was by participant self-report. Although, anecdotally participants appeared to have no difficulties in remembering or communicating this information, it is possible that there may have been inaccuracies. In the current study, objective clinical data on use of disease modifying therapies, relapse severity/frequency, neurological disability (EDSS[27], date of conversion from RRMS to SPMS or speed of progression was not systematically collected. However, it did seem that pwMS’s own memories and impressions of the frequency and severity of disease activity strongly influenced their experience of their own disease to date, and that this fed into their expectations of the future. Exploration of the relationships between a wide range of clinical characteristics and the prognosis-related beliefs, expectations, experiences and preferences of pwMS could be a valuable direction for future research.

Finally, this study did not explicitly raise the prospect of newly-emerging prognostication tools (e.g. OLAP [16]) with participants. Detailed studies of pwMS’s interest in and willingness to use these tools and their impact on decision-making and psychological adjustment will be another important step towards understanding how beliefs and communication about prognosis affect the health and wellbeing of pwMS.

## Supporting Information

S1 Fig Interview Schedule. (DOCX)
