# Supplementary material for: How Do People with Multiple Sclerosis Experience Prognostic Uncertainty and Prognosis Communication? A Qualitative Study
Source: PLoS One. 2016 Jul 19;11(7):e0158982. doi: 10.1371/journal.pone.0158982 (PMC4951148; doi:10.1371/journal.pone.0158982)
Supplement: S1 Fig — (DOCX) [file pone.0158982.s001.docx]

S1 Fig

**Supplementary Information: Interview Schedule**

1. I want to ask you to think back to the time when you were first diagnosed with MS. What do you remember being told early on about how your MS might affect you over the longer term?
   1. Can you tell me about how you feel about
      1. What you were told
      2. How you were told
      3. Tell me about anything that could have gone better
2. Can you remember any other/later occasions that your neurologist, MS nurse or GP talked to you about how your MS might affect you over the longer term
3. Can you tell me how you feel about
   - 1. What you were told
     2. How you were told
     3. Tell me about anything that could have gone better
4. How have your ideas about how MS is going to affect you changed or developed over the months or years since your diagnosis?

4. Can I ask you about your current understanding of how your MS might affect you over the longer term? What are your beliefs or expectations about this?

a. Who or what has influenced what you think or believe about this?

b. Is there anything or anyone else that has been important in forming this view?

1. How has your understanding about (or your uncertainty about) the likely long-term course of your MS affected you?
   1. Can you tell me about any ways that it has been helpful?
   2. Can you tell me about any ways that it has been unhelpful?
2. Can you tell me about whether trying to get detailed or specific information about how your MS might progress is something important for you, personally, at the moment?
   1. Can you expand on what makes you feel like that?
3. Who or what do you think could make things clearer for you?

Any ideas of what sort of things would help you?

1. In your opinion, what should health professionals think about when they talk to people with MS about how the disease might affect them in the future?

Standard prompts for each question/sub question:

- Can you tell me more about that?
- Can you expand on that?
- How did/do you feel?
- What was/is that like for you?
